# A Community Needs Assessment and Implementation Planning for a Community Exercise Program for Survivors of Stroke: Protocol for a Pilot Hybrid Type I Clinical Effectiveness and Implementation Study

**DOI:** 10.2196/55432

**Published:** 2024-04-11

**Authors:** Elizabeth Wherley Regan, Pamela Toto, Jennifer Brach

**Affiliations:** 1 Department of Exercise Science Arnold School of Public Health University of South Carolina Columbia, SC United States; 2 Department of Occupational Therapy School of Health and Rehabilitation Sciences University of Pittsburgh Pittsburgh, PA United States; 3 Department of Physical Therapy School of Health and Rehabilitation Sciences University of Pittsburgh Pittsburgh, PA United States

**Keywords:** community participatory research, need assessment, exercise, survivors of stroke, community, community need, exercise program, stroke, physical activity, PA, mobility impairments, impairment, mobility, health decline, group activity, group exercise

## Abstract

**Background:**

Physical activity and exercise are important aspects of maintaining health. People with mobility impairments, including survivors of stroke, are less likely to exercise and at greater risk of developing or worsening chronic health conditions. Increasing accessible, desired options for exercise may address the gap in available physical activity programs, provide an opportunity for continued services after rehabilitation, and cultivate social connections for people after stroke and others with mobility impairments. Existing evidence-based community programs for people after stroke target cardiovascular endurance, mobility, walking ability, balance, and education. While much is known about the effectiveness of these programs, it is important to understand the local environment as implementation and sustainment strategies are context-specific.

**Objective:**

This study protocol aims to evaluate community needs and resources for exercise for adults living with mobility impairments with initial emphasis on survivors of stroke in Richland County, South Carolina. Results will inform a hybrid type I effectiveness and implementation pilot of an evidence-based group exercise program for survivors of stroke.

**Methods:**

The exploration and preparation phases of the EPIS (Exploration, Preparation, Implementation, and Sustainment) implementation model guide the study. A community needs assessment will evaluate the needs and desires of survivors of stroke through qualitative semistructured interviews with survivors of stroke, rehabilitation professionals, and fitness trainers serving people with mobility impairments. Additional data will be collected from survivors of stroke through a survey. Fitness center sites will be assessed through interviews and the Accessibility Instrument Measuring Fitness and Recreation Environments inventory. Qualitative data will be evaluated using content analysis and supported by mean survey results. Data will be categorized by the community (outer context), potential participants (outer context), and fitness center (inner context) and evaluate needs, resources, barriers, and facilitators. Results will inform evidence-based exercise program selection, adaptations, and specific local implementation strategies to influence success. Pilot outcome measures for participants (clinical effectiveness), process, and program delivery levels will be identified. An implementation logic model for interventions will be created to reflect the design elements for the pilot and their complex interactions.

**Results:**

The study was reviewed by the institutional review board and exempt approved on December 19, 2023. The study data collection began in January 2024 and is projected to be completed in June 2024. A total of 17 participants have been interviewed as of manuscript submission. Results are expected to be published in early 2025.

**Conclusions:**

Performing a needs assessment before implementing it in the community allows for early identification of complex relationships and preplanning to address problems that cannot be anticipated in controlled effectiveness research. Evaluation and preparation prior to implementation of a community exercise program enhance the potential to be successful, valued, and sustained in the community.

**International Registered Report Identifier (IRRID):**

DERR1-10.2196/55432

## Introduction

Physical activity (PA) and exercise are important aspects of maintaining health and reducing the development and severity of chronic conditions [1]. PA can lower the risk of mortality and reduce the incidence of hypertension, diabetes, heart disease, and some cancers [1]. Older adults have additional benefits of improved quality of life and lower risk of falls [1]. People living with disability are at a greater risk for chronic health conditions and are less likely to exercise than their age-matched peers [2,3]. People with disabilities have limited access to exercise in order to mitigate their health conditions and improve their aerobic capacity [2,4].

For survivors of stroke, reduced PA additionally increases their risk for a recurrent stroke [3]. Up to 80% of the risk of recurrent stroke can be mediated by lifestyle factors including medication management, diet, and exercise [3]. Individuals after stroke with mobility impairments have poor movement economy and higher cardiovascular demands that increases their need to build aerobic capacity over their peers [5]. However, even after receiving inpatient rehabilitation services, deficits remain. After completing inpatient rehabilitation, 80% of stroke survivors can ambulate indoors, but only 27% can perform the essential skills of community ambulation [6]. Despite the remaining deficits, many do not receive additional care in the community [7]. A lack of services after rehabilitation to promote independent community living results in unmet needs for continued physical recovery [4,6,8].

In addition to the physical benefits of PA, structured exercise programs can provide social connection [9,10]. People living with disability, including survivors of stroke, are socially isolated and desire a sense of belonging and social participation [7,10,11]. Increasing accessible, desired options for exercise may address the gap in available PA programs, provide an opportunity for continued services after rehabilitation, and cultivate social connections for people after stroke and others with mobility impairments. Group exercise programs that focus on functional fitness for clinical populations are a worldwide fitness trend and are a suggestion of focus for fitness professionals by the American College of Sports Medicine [12].

Existing evidence-based community programs for people after stroke include Fitness and Mobility Exercise [13], Together in Movement and Exercise [14], and Fit for Function [15]. These programs target fitness with a varied focus on cardiovascular endurance, mobility, walking ability, balance, and education [13-15]. While much is known about the effectiveness of these programs, it is important to understand the local environment as implementation and sustainment strategies are context-specific.

The aim of this study protocol is to evaluate community needs and resources for general and group exercise for adults living with mobility impairments with initial emphasis on survivors of stroke in Richland County, South Carolina. Results will inform an action plan for a hybrid type I implementation study testing the effectiveness and implementation process of a modified evidence-based exercise intervention [16]. Involving the community in the needs assessment and performing the needs assessment with a systematic approach facilitates the success and sustainability of the program [17,18].

## Methods

### Overview

This project will address the study objective using the EPIS (Exploration, Preparation, Implementation, and Sustainment) framework, concepts of community-based participatory research, and the implementation research logic model [17-19]. The exploration and preparation stages of EPIS seek to determine health needs, identify barriers and facilitators, select an evidence-based program, determine program modifications, and create implementation strategies [17]. These stages are the focus of this protocol. The EPIS implementation and sustainment stages test the evidence-based program and the ability of the program to be maintained in the community after the research process ends [17]. The EPIS framework considers participants inside and outside the organization (inner and outer context) that will offer the community program, the academic and community partners that bridge these contexts, and the characteristics of the program itself [17]. Each stage is dependent on and related to the others [17].

**Figure 1:**
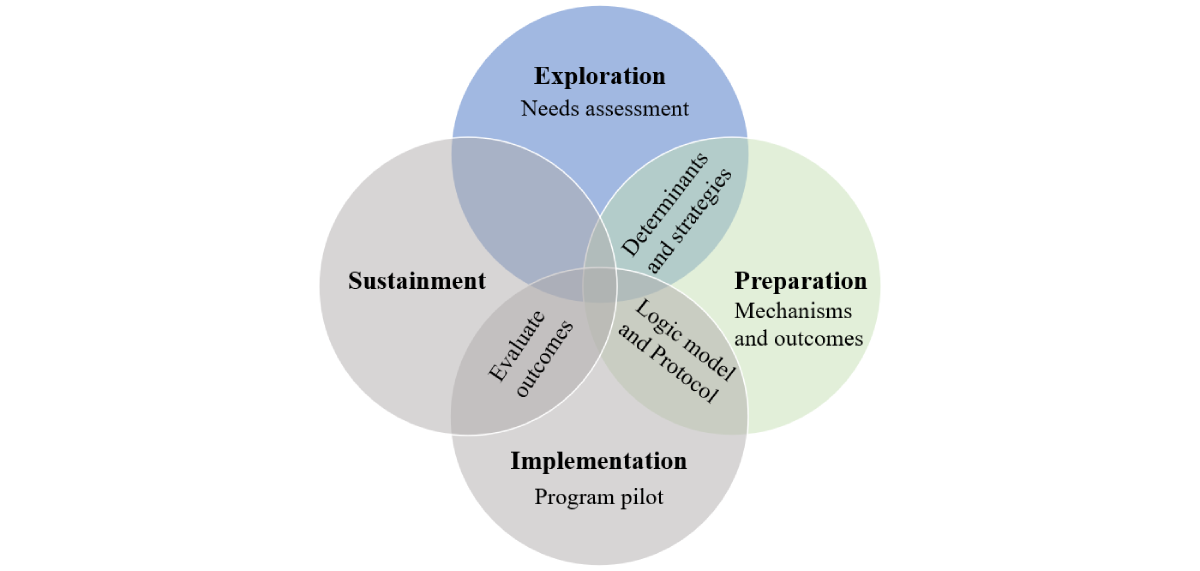
Applied EPIS (Exploration, Preparation, Implementation, and Sustainment) framework for implementation studies (adapted from Moullin et al [17], which is published under Creative Commons Attribution 4.0 International License [20]). The exploration and preparation stages will be completed as part of the current needs assessment for community group exercise for people after stroke. The exploration stage performs a needs assessment whose resulting determinants and strategies feed into the preparation stage that results in an implementation logic model and detailed protocol for the pilot study. The implementation and sustainment stages will be completed as a separate study and perform the pilot, evaluate the outcomes, and produce changes and further research for sustainment in the community.

**Figure 2:**
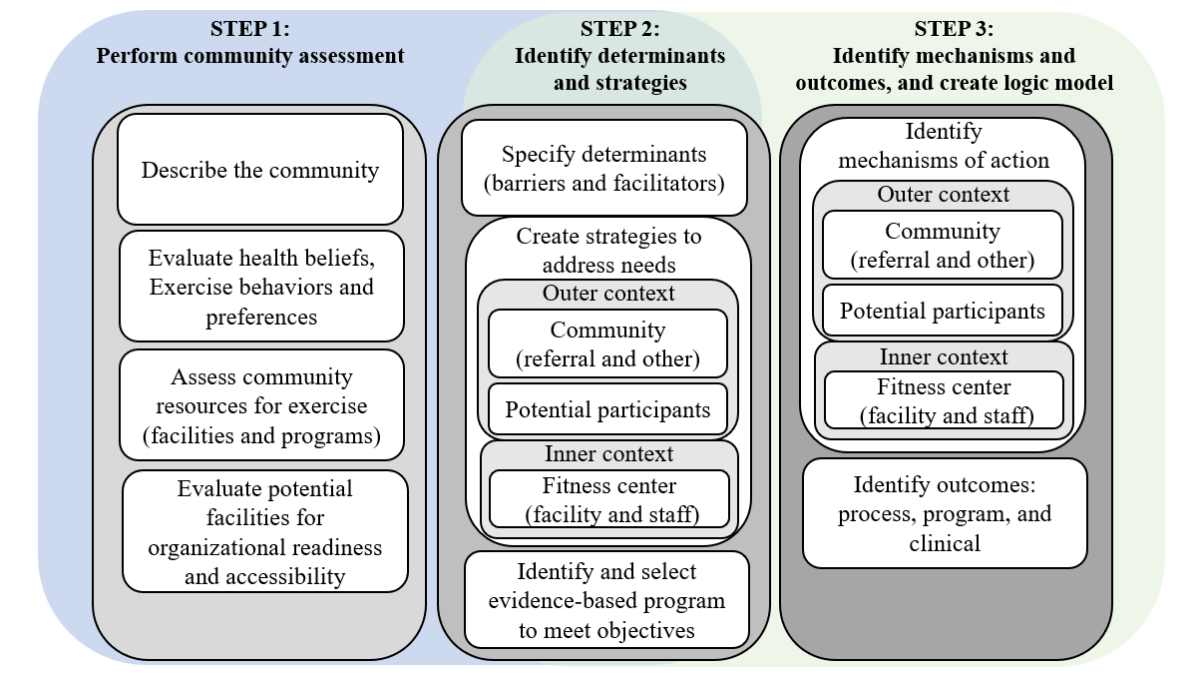
Steps to complete EPIS (Exploration, Preparation, Implementation, and Sustainment) framework: evaluation (blue-textured) and preparation stages (green-solid). This framework will be used as part of the needs assessment for a group exercise program for people after a stroke. Step 1 identifies the actions and data analysis of this study community needs assessment. Step 2 evaluates the results and selects the evidenced-based exercise program. Step 3 details the plans for pilot implementation including the mechanisms of action and the outcome measures.

The EPIS exploration and preparation stages (Figures 1 and 2) will be completed as part of this study and include three steps: (1) performing a needs assessment; (2) identifying determinants, selecting an evidence-based exercise program to address, and creating implementation strategies; and (3) determining mechanisms of action for the implementation strategies and their corresponding outcome measures, and creating a logic model [17,19]. Results will inform the implementation protocol and program pilot-testing as a separate study (EPIS implementation and sustainment stages; Figure 1 [17,20]).

### Step 1: Perform Community Assessment

#### Describe the Community

A literature review will provide perspective on the communities of Richland and Lexington Counties in South Carolina. Several resources will help provide demographics including United States Census data, Center for Disease Control American Community Survey and Center for Health Statistics, the Behavioral Risk Factor Surveillance System, and County Health Rankings. Prisma Health, the largest nonprofit health system serving Richland County SC, completed a community needs assessment in 2022. This assessment will provide data to describe the community health concerns and overall health priorities. General demographics, demographics of people living with disabilities, and demographics of survivors of stroke will be described.

#### Evaluate Health Beliefs, Exercise Behaviors, and Preferences

This step will use semistructured interviews with key stakeholders to assess the culture of the community around health beliefs and exercise behaviors and preferences both for people with mobility impairments and specifically for survivors of stroke. Individual or small group interviews will be conducted with targeted key organizational stakeholders which will include health care providers (physicians, rehabilitation providers, and cardiac rehabilitation providers), other nonprofit or public organizations serving people with disabilities (independent living support services, diabetes educators, vocational rehabilitation, and veterans’ organizations) and fitness providers who currently serve people with mobility issues. After initial contact with stakeholders in each category, snowball sampling [21] will identify additional people or organizations to include.

Interview questions will relate to health needs, specific exercise needs, beliefs, contextual factors, exercise services, and gaps. Survivors of stroke will be interviewed in a focus group format at a community stroke survivor support group and additionally in small groups. Care partners will be invited to participate. Interview questions will be the same regardless of format or stakeholder (Textbox 1). Questions will be piloted in the first 2 interviews and revised as needed.

Sample goals are to collect data from a diverse set of viewpoints in order to adequately answer study aims [22]. A pragmatic approach targets 10-15 stakeholders and 10-15 survivors of stroke for the initial sample to achieve.

In addition to the questions in Textbox 1, the stroke survivors focus group will receive additional questions related to specific desires around group exercise (Textbox 2).

Once the focus groups and individual interviews are complete, a survey will be created based on the results. The survey will be distributed to a wider audience in the community and will evaluate desires for general and group exercises and the barriers and facilitators to participation. The survey will be distributed through stakeholder organizations and research contact lists for people with mobility impairments using REDCap (Research Electronic Data Capture) electronic data capture tools hosted at the University of South Carolina.

**Semistructured interview questions for stakeholders to evaluate community health services and exercise beliefs and behaviors as part of the community needs assessment for a group exercise program for people after stroke. Health care providers, organizations serving people with disabilities, fitness professionals, survivors of stroke, and care partners will be interviewed in individual, small group, or focus group formats.**

**Questions:**

1. What is important to this community from a health and quality of life perspective?
2. What are the beliefs about access and treatment for ongoing health care needs?
3. What existing organizations and services address health care and quality of life needs?
  What are they doing well?
  What are the gaps in services?
4. What are the beliefs about and interest in exercise for this community?
5. What are the barriers and facilitators to exercise?
6. What are existing community resources for people to exercise and to engage in other ways to improve health?
  Who is using these resources? Describe the people who engage.
  What are they doing well?
  What are the gaps in services?
7. What are your thoughts on funding to support exercise programming on-going? (organizational stakeholders)?

**Semistructured interview questions to evaluate specific exercise interests and preferences for survivors of stroke only as part of the needs assessment for planning a community group exercise program for people after stroke. Interviews will occur in a focus group or small group format.**

**Semistructured interview questions on exercise interest and preferences (stroke survivors only):**

1. What types of exercise activities would you be interested in (endurance, strength, balance/coordination, stretching, and relaxation) Examples given of each type of exercise.
2. In what formats (small group, larger group/Type of group based on mobility: seated vs standing, requiring assistance vs nonrequiring assistance, based on diagnosis or not based on diagnosis)
3. What type of facility (community fitness facility, private gym, community center, senior center, church center) and in what part of the county is desired?
4. How often and what time of day is preferred?
5. What do you think the benefits of an exercise program would be for you individually and others with stroke?
6. What do you think you will like about exercising in a group? What do you think you will dislike about group exercise?
7. What are factors currently and potentially impacting access and engagement in exercise?
  Are you currently exercising, and if so, what are you doing, and if not, why not?
  What do you like about exercise or movement?
  What is your confidence in your ability to exercise independently? With instruction? With assistance?
    If low confidence, what do you think would increase confidence?
  How would you be able to get to a group exercise program (transportation)?
  How much would you be willing to pay for an exercise program?

#### Assess Community Resources for Exercise

Using results from the community assessment and internet searches, the facilities and program resources available for group and individual exercise for people with mobility impairments in Richland County will be identified. Information gathered will include whether the facility or organization self-identifies as accessible and the goals and mission of the organization.

#### Evaluate Potential Facilities for Accessibility and Organizational Readiness

A subset of the listing of community resources will be evaluated in more detail to assess accessibility and general organizational readiness for new or modified exercise programs for people with mobility impairments (Textbox 3). Facility management, staff, and fitness providers will be interviewed to determine readiness for change. Interview questions were developed based on the components of the theory of organizational readiness for change [23]. The evaluation will include an accessibility evaluation using the Accessibility Instrument Measuring Fitness and Recreation Environments (AIMFREE) inventory [24].

**Potential location for pilot group exercise program for people after stroke facility evaluation will include facility stakeholder (exercise instructors and management) semistructured interviews in individual or small group format and a comprehensive accessibility inventory.**

Methods: (1) Stakeholder interview and (2) Accessibility Instrument Measuring Fitness and Recreation Environments (AIMFREE) accessibility inventory (Rimmer et al [24])

**Facility stakeholder interview questions:**

1. Describe the interest and priority in the organization to serve those with mobility impairments.
2. What is your openness to adding additional or modified programs for people with mobility impairments?
3. Describe your perception of your organization’s ability to support an exercise program for people with mobility impairments both initially and ongoing.
4. What are the resources available to support new programming?
  Describe your staff who could potentially support these new programs.
  Would new staff need to be hired?
  What are your anticipated barriers or facilitators?
  What types of equipment and space would be available? At what time of day?

**AIMFREE accessibility inventory:**

The inventory includes both physical space accessibility and behavior and policy evaluations. The AIMFREE inventory can be obtained from the National Center on Health, Physical Activity and Disability (NCHPAD), nchpad.org. The following sections will be completed with the assistance of facility staff and a consultant with mobility impairments. Section E, which evaluates hot tubs, whirlpools, and saunas will be omitted.

- SECTION A: Access Routes and Entrance
- SECTION B: Equipment
- SECTION C: Information
- SECTION D: Locker Rooms and Showers
- SECTION F: Elevators
- SECTION G: Bathrooms
- SECTION H: Professional Behavior
- SECTION I: Professional Support and Training
- SECTION J: Policies
- SECTION K: Programs

### Step 1: Data Analysis

#### Outer Context Description

A narrative summary of the neighborhood characteristics and priorities in the community based on the formal community statistics and health priorities review will be created to provide descriptive background for the remaining data. Community participants will be described with demographic descriptive statistics for each group (referral sources, health care providers, fitness providers, community organizations, and potential participants).

#### Inner Context Description

General descriptions of fitness facilities including location, program availability, inclusion as part of the mission, and goals of the organizations in Richland County will be summarized. For the subset of facilities that receive detailed reviews, a summary of each facility will be provided. The summary will include the AIMFREE findings.

#### Qualitative Thematic Analysis and Survey Analysis

Focus group and interview data will be analyzed using deductive-dominant qualitative content analysis using NVivo software (version 12; Luminvero Inc) [25-27]. Captured data will be open-coded and categorized for thematic analysis in the following levels: community (outer context), individual (outer context), and fitness center (inner context). At each level, needs/desires, resources, barriers, and facilitators will be defined by the responses to the categories in Table 1 by 2 researchers and combined by group review and discussion to resolve discrepancies. Any themes newly arising from the data outside of these categories will be included. Survey results will also be mapped to the same categories from Table 1 and combined in an evaluation matrix with the themes from the content analysis.

**Table 1:** Categories of determinants and implementation strategies for results in the outer context and the inner context of the needs assessment for group exercise for people after stroke. Determinants categories will serve as the organizational structure for qualitative thematic data analysis and implementation strategies categories will serve as the planning structure for the implementation protocol.

Categories and levels		Determinants categories	Implementation strategies categories
**Outer context**			
	Community	Health care and other potential referral resources and the facilitators and barriers to referral Other assistance needed from health care or community organizations to support potential new programs.	Identify referral sources Create strategies to address barriers to referral Build strategies to address needs for health care or other community supports vital to the success of the program including champions
	Potential participant	General health and quality of life priorities, services, and organizations to meet related needs and gaps in services. General exercise resources, use, beliefs, and priorities General exercise desires and needs Barriers and facilitators to exercise	Strategies to address barriers and promote uptake and retention of potential participants
**Inner context**			
	Intervention characteristics	Group exercise desired format, types of exercise, and frequency	Identify and select evidence-based exercise program
	Organizational (fitness facility)	Available facilities and programs already meeting the identified individual needs and desires Accessibility of targeted facilities/organization (physical, behavioral, policy) including gaps and potential future needs Organizational readiness for change	Select an organization to pilot the program Identify organizational strategies for staff, training, equipment, accessibility upgrades, and other needs Identify an organizational champion

### Step 2: Identify Determinants and Implementation Strategies

Following the EPIS framework, the preparation stage begins with specifying the determinants based on results in step 1. Determinants include contextual factors for the outer context (potential participants and community) and inner context (fitness facility), process components, and intervention components [19]. From the determinants, an evidenced-based exercise program will be selected, and implementation strategies for exercise programming and related services created (Figure 2 and Table 1) [19]. Implementation strategies are approaches to address the necessary modifications of the exercise program, the training and resources needed for the inner context, and any other required innovations to adapt to the local community.

All results of the qualitative content analysis, triangulation with survey data, and the determinants will be verified through member checking with updates made as necessary.

### Step 3: Identify Mechanisms, Outcomes, and Create Logic Model

**Figure 3:**
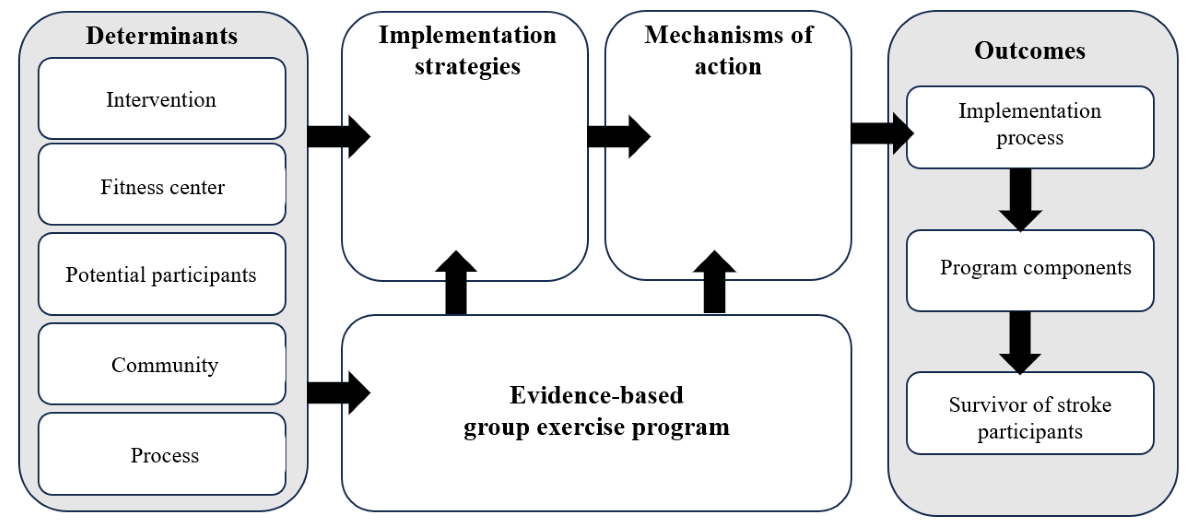
The implementation research logic model standard form with intervention (adapted from Smith et al [19], which is published under Creative Commons Attribution 4.0 International License [20]). The logic model will serve as a roadmap for the implementation protocol for the evidence-based group exercise program for people after a stroke. Determinants include contextual factors, process components, and intervention components. Implementation strategies are actions to address the unique determinants through mechanisms of action (ie, education and skill building) to ensure success. Outcomes are process, program, and participant-driven.

Identifying the mechanisms of action, selecting outcomes, and creating the logic model addresses the preparation step of the EPIS framework. Mechanisms of action are the implementation strategy processes to affect the outcomes [28]. Examples include education, skill building, efficacy, and motivation [28]. Finally, outcome measures for the participants (clinical effectiveness), implementation process (fidelity), and program components (service) will be identified. A logic model will be created based on the results of steps 2 and 3 to organize the determinants, implementation strategies, exercise programs, mechanisms of action, and outcomes. A logic model demonstrates the study design elements for the hybrid type I model and their complex interactions [19]. The standard logic model format by Smith et al [19] is presented in Figure 3 [19,20].

### Ethical Considerations

The study has been reviewed by the University of South Carolina Institutional Review Board and classified as exempt human participant research. Letters of information are provided to participants outlining the study. Data will be collected and stored in REDCap cloud storage or other password-protected cloud storage at the University of South Carolina. Transcripts will be deidentified prior to data analysis. Participants will be compensated with a US $25 gift card upon completion of interviews.

## Results

As of March 2024, the project is in process. The literature review has been completed. A total of 4 fitness professionals, 4 physical therapists, 1 physiatrist, 2 directors at Able South Carolina (a nonprofit community organization), 7 survivors of stroke, and 1 care partner have been interviewed. Two potential exercise facilities have been identified.

## Discussion

### Protocol Goals and Anticipated Outcomes

This protocol will evaluate the desires and needs of the local community for an evidence-based group exercise program for people after stroke and the planning required to implement the program. The use of community-based participatory research at the early stage of implementation planning will enhance the fit and success of the future community program [18]. Involving the community in the planning stages builds trust and identifies shared values toward a common goal [18]. The selection of the evidence-based program will be based on identified needs and desires [17]. The needs assessment will also provide a rationale for required modifications to the standard program [17]. If no evidence-based program meets the majority of the needs of the community, a unique program drawing on available evidence-based resources will be developed.

Performing a needs assessment before implementing it in the community allows for early identification of the complex relationships between the inner and outer contexts of a community program [17,19]. A result is an ability to preplan implementation strategies to address problems that cannot be anticipated in controlled effectiveness research. Capacity building, education and training, and recruitment and retention strategies will be identified and built into the action plan [17,19].

### Study Strengths and Limitations

Using the EPIS framework, implementation mapping processes and the implementation logic model are strengths of this protocol [17,19,29]. The use of EPIS and related practical application tools creates a systematic approach to a complex task [17,19,29].

Potential study limitations include difficulty finding willing participants, reduced completion of surveys, and difficulty matching an evidence-based program to the needs of the community. These potential pitfalls are mediated by the ability to leverage existing academic-community relationships, multiple sources of data collection, and the existence of multiple potential evidence-based programs to choose from. Existing evidence-based community programs for people after stroke or for older individuals may be adapted through this process to meet the community’s needs and desires.

### Conclusions

Performing thorough evaluation and preparation prior to the implementation of a community exercise program will enhance its ability to be successful, valued, and sustained in the community. A robust process that includes community partners, applied theory, and implementation process tools enhance the protocol.

## Abbreviations

AIMFREE: Accessibility Instrument Measuring Fitness and Recreation Environments
EPIS: Exploration, Preparation, Implementation and Sustainment
PA: physical activity
REDCap: Research Electronic Data Capture

## Data Availability

Data sharing is not applicable to this article as no data sets were generated or analyzed during this study
